# Public Beliefs and Perception of Mental Disorders in Poland—A 2025 Nationwide Cross-Sectional Survey

**DOI:** 10.3390/jcm14134586

**Published:** 2025-06-28

**Authors:** Aleksandra Lewandowska, Mateusz Jankowski, Mariusz Gujski, Aneta Duda-Zalewska, Piotr Jedrusik, Andrzej Silczuk

**Affiliations:** 1 Children Psychiatry Unit Specialized Psychiatric Health Care Centre in Lodz, 91-229 Lodz, Poland; 2 Department of Population Health, Centre of Postgraduate Medical Education, 01-826 Warsaw, Poland; 3 Department of Public Health, Medical University of Warsaw, 02-106 Warsaw, Poland; 4 Department of Internal Medicine, Hypertension and Vascular Diseases, Medical University of Warsaw, 00-575 Warsaw, Poland; 5 Department of Environmental Psychiatry, Faculty of Life Sciences, Medical University of Warsaw, 02-353 Warsaw, Poland

**Keywords:** mental health, mental disorders, prevention, school, public policy

## Abstract

**Background/Objectives**: Globally, one in eight people live with a mental disorder, with depression being a leading cause of disability. This study aimed to identify sociodemographic factors associated with public belief and perception of mental disorders among adults in Poland. **Methods**: A cross-sectional study with a self-prepared questionnaire (5-point Likert scale) was administered to a nationally representative quota sample of 1114 Polish adults (March 2025). The computer-assisted web interview (CAWI) method was applied. **Results**: A cross-sectional survey of 1114 adults found that 23.2% had visited psychiatrists, 15.9% psychotherapists, and 21.1% reported a family history of mental disorders. Most respondents (73.8%) believed employers are reluctant to hire individuals with mental disorders, and 53.8% perceived discrimination. Additionally, 19.4% thought mental health patients receive lower-quality care than those with physical illnesses like diabetes. Respondents from large cities (≥500,000 residents, aOR: 1.67; 95%CI: 1.11–2.51; *p* = 0.01), with higher education (aOR: 1.62; 95%CI: 1.26–2.07; *p* < 0.001), or a family history of mental disorders (*p* < 0.05) were more likely to hold this view. Higher education (aOR: 1.47; 95%CI: 1.11–1.94; *p* = 0.01), good economic status (aOR: 1.60; 1.06–2.40; *p* = 0.02), and personal psychiatric experience (aOR: 1.89; 95%CI: 1.24–2.87; *p* = 0.003) increased belief in treatment effectiveness. Males (aOR: 1.88; 95%CI: 1.36–2.61; *p* < 0.001) and medium-city residents (aOR: 1.82; 95%CI: 1.01–3.27; *p* = 0.04) more often perceived mental disorders as a sign of weakness, while women (aOR: 1.74; 95%CI: 1.36–2.22; *p* < 0.001) and those with affected relatives (*p* < 0.05) more frequently reported discrimination. Older respondents (*p* < 0.05), those with higher education (aOR: 1.65), and individuals with a family history of mental disorders (*p* < 0.05) were more likely to state that employers fear hiring people with psychiatric conditions. **Conclusions**: These findings underscore the need for public health interventions to reduce stigma, improve awareness, and address misconceptions about mental disorders in Poland.

## 1. Introduction

The World Health Organization (WHO) estimated that approximately one in eight people worldwide are living with a mental disorder, with anxiety and depressive disorders being the most common [1]. Depression is expected to be a major contributor to the global burden of disease by 2030. Depression currently causes more disability in young people aged 10 to 24 than any other disease [1]. People with mental disorders experience disproportionately higher rates of disability and mortality [2]. People with major depressive disorder and schizophrenia had a 40 to 60% higher risk of premature death than the general population [3]. Despite growing public awareness and education about mental health, people diagnosed with mental disorders continue to struggle with deep-rooted stigma [3,4,5]. Stereotypes, prejudices, and lack of understanding mean that instead of receiving support, they often experience isolation, exclusion, and discrimination—both in their social and professional lives. Stigma not only increases the suffering of those affected by the disease, but also discourages them from seeking help, complicates treatment, and negatively affects quality of life. People with mental health problems are three to seven times more likely to be unemployed than those without [4]. This is a critical issue, as employment opportunities help rebuild self-esteem and are an important part of the healing process [5]. Unemployment correlates with poorer overall health, including mental health, poverty, and a higher risk of suicide [6,7]. Employment brings numerous benefits to mental health, such as the structure of the day, a sense of purpose, daily activity, and greater financial security, protecting against stress resulting from insufficient income [8]. In turn, taking up a job in favorable conditions not only improves overall health, but also strengthens the sense of self-worth, agency, and happiness. It also supports the healing process of people who have experienced a mental crisis in many dimensions—functional, existential, and social [9].

One of the main barriers faced by people with mental disorders is stigma and discrimination in the workplace [10]. Both negative attitudes and behaviors on the part of employers, as well as the expected stigmatization and self-stigma of people diagnosed with mental disorders themselves, make it difficult for them to find and maintain employment [11]. For example, in one recent representative study, as many as 64% of Dutch managers expressed reluctance to hire a job applicant who was experiencing a mental crisis, and 30% declared resistance even to hiring a candidate who had already recovered [12]. Moreover, it has been shown that the experience of discrimination related to a mental disorder can negatively affect motivation and job search activities.

A study by Thornicroft et al., who documented the personal experiences of the stigma of 732 people with schizophrenia from 27 developed and developing countries, found that as many as three-quarters (72%) of the sample indicated that they felt a need to conceal their diagnosis, 64% expected discrimination when applying for work, training or education, and 55% anticipated discrimination in close relationships [13]. The effects of discrimination were evident in a wide range of everyday experiences, such as interactions with family, friends, and employers, in all countries studied. Seeman et al. conducted a global study of the stigma of mental disorders using an innovative online platform that reached over half a million respondents from 229 countries [14]. Although the study was broad in scope, it did not use a standardized scale to measure stigma, and the profile of participants was likely limited to those familiar with technology—mainly young, educated men. In highly developed countries such as Canada, the United States, and Australia, only 7–8% of respondents considered people with mental disorders to be more aggressive [14]. Meanwhile, in developing countries such as Algeria, Mexico, Morocco, and China, this percentage increased to 15–16% [14]. The authors suggest that these differences may be influenced by cultural factors, tradition, and the level of education and access to healthcare—elements that shape the social perception of mental health [14]. People diagnosed with mental disorders are often excluded from society and discriminated against by others. The main factor that determines the belonging of such people to the community is social policy. Inappropriate attitudes may also result from individual factors—fear of otherness, fear of independence and dependency, lack of knowledge and personal experience in contact with such people, and inability to behave in their company [15]. Stigmatization of people diagnosed with mental disorders is not only an individual problem—it is a social problem that requires empathy, knowledge, and action. The more we raise the topic of mental health in the public space, the greater the chance that people who need help will be able to reach for it without fear and shame.

Nationwide data on public beliefs and perceptions of mental disorders may be used to improve mental health care programs and services. The stigma of people with mental disorders may lead to delayed diagnosis or even to avoiding visits to healthcare professionals [10,11]. Moreover, data on social perception of mental disorders may inform healthcare professionals of patients’ perspectives on mental health and provide useful information that can be used in physician–patient communication on mental health issues. The burden of mental disorders in Poland is relatively high [16,17]. Moreover, there are numerous actions on mental health in Poland, including new types of health services, reorganization of mental health facilities, and educational campaigns on mental health and mental health literacy [1,16,17]. However, there is a lack of representative data on public perception of mental disorders and their impact on the social and professional life of people with mental disorders.

This study aimed to identify sociodemographic factors associated with public belief and perception of mental disorders among adults in Poland.

## 2. Materials and Methods

### 2.1. Study Design

This cross-sectional study was conducted between 8 and 10 March 2025, utilizing the computer-assisted web interview (CAWI) technique for data collection. A structured questionnaire (Supplementary Material S1), developed by the research team, was administered to a nationally representative sample of adults in Poland. To ensure methodological rigor and data quality, a professional public opinion research agency (Arianda Poland’s Research Panel [18]) was contracted to collect data. The authors provided the scientific framework and designed the research instrument. The questionnaire was made available online via a secure research platform managed by the contracted agency. Each selected respondent received a personalized invitation via email, followed by a reminder text message. Each respondent was obligated to answer all the questions, so there were no missing answers. The overall response rate was estimated at 22%.

### 2.2. Population

The study sample was drawn from a verified panel of over 100,000 registered respondents. A stratified quota sampling approach was applied to ensure representativeness, based on key demographic characteristics including gender, age, and place of residence, with national statistical data published by Statistics Poland [19]. If an initially selected participant declined to participate, the next eligible individual meeting the stratification criteria was invited.

The methodological framework applied in this study (a nationwide cross-sectional survey) aligns with previously conducted nationwide cross-sectional surveys in Poland [20,21], ensuring population inference.

Participation in this study was voluntary and anonymous. Ethical approval of the Ethics Committee of the Medical University of Warsaw was obtained (decision AKBE/38/2025, as of 24 February 2025) and the study followed the Helsinki Declaration rules.

### 2.3. Study Questionnaire and Measures

The study questionnaire was based on a literature review [10,11,12,13,14,15]. A group of physicians, psychiatrists, and public health specialists reviewed the questionnaire. A group of eight adults completed the questionnaire twice, five days apart, within the pilot program. After this pilot program, two questions were revised to better align with the respondents’ perception and reduce the risk of bias.

Five different questions with a 5-point Likert response scale were addressed to assess public beliefs and perceptions of mental disorders in Poland (Supplementary Material S1). For the purposes of statistical analysis, in four questions, responses “rather yes” or “definitely yes” were combined into one variable. Moreover, in one question, the responses “rather no” or “definitely no” were combined into one variable.

### 2.4. Statistical Analysis

IBM SPSS version 29 (Armonk, NY, USA) was used to run statistical analysis. Frequencies and percentages of the categorical variable distribution were displayed. Categorical variables were compared using cross-tables and chi-squared testing.

Factors associated with public beliefs and perceptions of mental disorders were identified using logistic regression analyses. Five different series of analyses were prepared. Every sociodemographic variable was considered independently in bivariable logistic regression. Every variable shown to be statistically significant in the bivariable analysis was incorporated into the multivariable logistic model. The odds ratio (OR) and 95% confidence intervals (95%CIs) were used to show the strength of the associations. The statistical significance level was set at *p* < 0.05.

## 3. Results

### 3.1. Study Population

Characteristics of the study population are presented in Table 1. Among the respondents, 23.2% had visited psychiatrists and 15.9% had visited psychotherapists. A history of mental disorders in close family (parents, children, spouse) was declared by 21.1% of respondents, and 10.4% of respondents declared a history of mental disorders in close friends (Table 1).

### 3.2. Public Beliefs and Perception of Mental Disorders

Only 19.4% of respondents (4.2% definitely yes and 15.2% rather yes) declared that people with mental disorders) in Poland receive the same level of care as people with physical diseases like diabetes or hypertension (Table 2). Most of the respondents believed that mental disorders can be effectively treated (17.2% of respondents stated definitely yes and 55.7% rather yes). Only 16.2% of respondents (3.3% of respondents stated definitely yes and 12.9% rather yes) believed that mental disorders are a sign of weakness (Table 2). Over half of the respondents (11.7% of respondents stated definitely yes and 41.8% rather yes) declared that people diagnosed with mental disorders are discriminated against in Poland. Among the respondents, 73.8% (26% of respondents stated definitely yes and 47.8% rather yes) believed that employers are afraid to hire people diagnosed with mental disorders (Table 2).

### 3.3. Sociodemographic Differences in Public Beliefs and Perception of Mental Disorders

Respondents who lived in cities ≥ 500,000 residents (aOR: 1.67; 95%CI: 1.11–2.51; *p* = 0.01), those with higher education (aOR: 1.62; 95%CI: 1.26–2.07; *p* < 0.001), as well as those with a history of mental disorders in close family or close friends (*p* < 0.05), were more likely to declare that people with mental disorders do not receive the same level of care as people with physical problems like diabetes (Table 3).

Respondents with higher education (aOR: 1.47; 95%CI: 1.11–1.94; *p* = 0.01), those with good self-reported economic status (aOR: 1.60; 1.06–2.40; *p* = 0.02), those who had ever visited a psychiatrist due to their own mental health issues (aOR: 1.89; 95%CI: 1.24–2.87; *p* = 0.003), as well as those with a history of mental disorders in close family or close friends (*p* < 0.05), were more likely to declare that mental disorders can be effectively treated (Table 4).

Males (aOR: 1.88; 95%CI: 1.36–2.61; *p* < 0.001) and those who lived in cities from 20,000 to 99,999 residents (aOR: 1.82; 95%CI: 1.01–3.27; *p* = 0.04), were more likely to perceive mental disorders as a sign of weakness (Table 5).

Females (aOR: 1.74; 95%CI: 1.36–2.22; *p* < 0.001), as well as those with a history of mental disorders in close family or close friends (*p* < 0.05), were more likely to declare that people with mental disorders are discriminated against in Poland (Table 6).

Respondents aged 50 and over (*p* < 0.05), those with higher education (aOR: 1.65; 95%CI: 1.25–2.19; *p* < 0.001), as well as those with a history of mental disorders in close family or close friends (*p* < 0.05), were more likely to declare that employers are afraid to hire people diagnosed with mental disorders (Table 7).

## 4. Discussion

This study provides nationwide data on public beliefs and perceptions of mental disorders in Poland. Most adults believe that mental disorders can be effectively treated, but less than one-fifth declared that people with mental disorders in Poland receive the same level of care as people with physical diseases like diabetes or hypertension. Most adults in Poland do not perceive mental disorders as signs of weakness, but people with mental disorders are considered as those who experience discrimination. In addition, there is a belief that employers are afraid to hire people diagnosed with mental disorders. In this study, significant differences in public beliefs and perceptions of mental disorders were observed, with the history of mental disorders in close family or close friends as the most important factor associated with public perception of mental disorders.

In recent years, Poland has launched the WHO Mental Health Gap Action Programme (mhGAP), an evidence-based approach to scaling up capacity and services for mental health conditions, under the National Health Programme [22]. The current reform of the public psychiatric care system in Poland aims to provide the necessary human and infrastructural resources to meet the health needs of Poles in the field of mental health services [22]. In Poland, under mandatory health insurance, all insured have access to mental health services, and a psychiatrist is listed as one of five medical specialists that can be visited without referral [23]. Despite the ongoing changes in psychiatric care in Poland, only one-fifth of respondents believed that people with mental disorders (e.g., depression) in Poland receive the same level of care as people with physical problems (e.g., diabetes or hypertension). This observation suggests that health needs related to mental care services are not sufficiently addressed in Poland. Respondents who lived in cities ≥ 500,000 residents, as well as those with higher education, were more likely to declare that people with mental disorders do not receive the same level of care as people with physical problems like diabetes. This observation may suggest that even in big cities, barriers to access to psychiatric care are presented. Moreover, respondents with a history of mental disorders in close family or close friends were more likely to declare that people with mental disorders do not receive the same level of care as people with physical problems like diabetes, which may result from the fact that those people experienced difficulties in access to psychiatric care among people from the immediate environment.

In this study, 73.8% of respondents believed that mental disorders can be effectively treated. This observation suggests that, despite perceived barriers to access to mental health, most adults in Poland believe in the effectiveness of psychiatric care in Poland. Numerous groups of patients with mental disorders can be effectively treated and remain in remission with, e.g., psychotherapies and/or pharmacotherapies [24,25]. Respondents with higher education, as well as those with good economic status, were more likely to declare that mental disorders can be effectively treated. This observation may result from the fact that these sociodemographic groups may represent higher levels of mental health literacy [26,27]. Moreover, respondents who had visited psychiatrists due to their mental health issues and those with a history of mental disorders in close family or close friends were more likely to declare that mental disorders can be effectively treated. This observation may result from the fact that those who used psychiatric care or have close relationships with those who used psychiatric care saw positive effects of psychiatric care and are aware of possibilities resulting from the current therapeutic methods used in psychiatry [26,27].

In this study, only a small section of respondents (16.2%) perceived mental disorders as signs of weakness, with males and inhabitants of cities from 20,000 to 99,999 residents as groups that were more likely to perceive mental disorders as a sign of weakness. This observation points out a need to target males and inhabitants of medium-sized cities (from 20,000 to 99,999 residents) as priority groups for educational campaigns on mental disorders, their causes, and health and social consequences. Anti-stigma programming is an important part of public health actions, and may shape public attitudes toward mental disorders in different cultural and social settings [28].

People with mental disorders may experience significant health disparities and stigma [29,30]. Addressing mental health stigma is a significant part of public policies on mental health [30]. In this study, 53.8% of respondents declared that people with mental disorders are discriminated against in Poland. Females and those with a history of mental disorders in close family or close friends were more likely to declare that people with mental disorders are discriminated against. This observation suggests that mental disorders remain as both health and social issues, as people with mental disorders may experience discrimination. Building mental health literacy may help to reduce mental health stigma [31]. Psychiatric clerkship was shown as an effective tool to reduce stigmatizing attitudes toward mental disorders [32]. Human storytelling and the involvement of public bodies in educational and anti-stigma campaigns should be considered as a part of the public campaigns on mental health disorders.

People with mental health problems are three to seven times more likely to be unemployed than those without mental disorders [4]. In this study, almost three-quarters of respondents believed that employers are afraid to hire people diagnosed with mental disorders. Public authorities should work on legal rules that secure the needs of employees who experience mental health deterioration. Stigma awareness intervention was also described as an effective intervention that allows reemployment of people with mental disorders [33]. Respondents aged 50 and over, those with higher education, and those with a history of mental disorders in close family or close friends were more likely to declare that employers are afraid to hire people diagnosed with mental disorders. We can hypothesize that the abovementioned groups may know people who experienced difficulties with the employment of people with mental disorders.

The are several practical implications resulting from this study. Adults in Poland believe that people with mental disorders do not have sufficient access to health services, so actions are needed to increase the capacity of mental health services in Poland. People with mental disorders are perceived as those exposed to discrimination, so educational campaigns are needed to build public awareness of mental disorders and shape evidence-based public attitudes towards mental disorders. Educational campaigns should be particularly targeted to males, those without higher education, and inhabitants of cities from 20,000 to 99,999 residents. There is an also need to educate healthcare professionals on how to talk with patients and their relatives, in a way that provides comfort and reduces risk of stigma. Moreover, policymakers should develop policies and stigma reduction strategies that will ensure job security and stable employment for people with mental disorders and will reduce the phenomenon of unemployment caused by employers’ lack of willingness to employ people with mental disorders.

This cross-sectional survey has several limitations. First, the CAWI method was used, so only respondents with internet access could participate (although 96% of households in Poland have internet access [34]) in this survey. There was no direct contact with the respondents, and they could not ask questions to the research team, so recall bias may occur. Assessment of public beliefs and perception of mental disorders was limited to five key questions. Further studies may include a broader scope of questions. The history of visits to psychiatrists or psychotherapists, as well as the history of mental disorders in close family or friends, were self-declared and medical documentation was not verified. In this study, the data analysis approach was like that of previously published studies, with the same data collection method [20,35]. However, we did not perform model fit statistics (e.g., Hosmer–Lemeshow, ROC curves), nor did we apply correction for multiple comparisons, as the study aimed at descriptive mapping and hypothesis generation.

## 5. Conclusions

Findings from this study revealed that people with mental disorders are perceived by society as those who do not have sufficient access to health services and are exposed to the risk of discrimination in society and difficulties in the labor market. A history of mental disorders in close family or close friends was the most important factor associated with public perception of mental disorders. Public health interventions are needed to address gaps and unjustified beliefs in the level of knowledge of mental disorders among adults in Poland.

## Figures and Tables

**Table 1:** Characteristics of the study population (*n* = 1114).

Variable	*n*	%
Gender		
female	609	54.7
male	505	45.3
Age [years]		
18–34	260	23.3
35–49	321	28.8
50–64	342	30.7
65+	191	17.1
Place of residence		
rural area	423	38.0
city below 20,000 residents	143	12.8
city from 20,000 to 99,999 residents	218	19.6
city from 100,000 to 499,999 residents	187	16.8
city ≥ 500,000 residents	143	12.8
Having higher education		
yes	508	45.6
no	606	54.4
Professional status		
active (employed or self-employed)	685	61.5
passive (unemployed, student or pensioner)	429	38.5
Married		
yes	612	54.9
no	502	45.1
Having children		
yes	727	65.3
no	387	34.7
Self-reported economic status		
good	524	47.0
moderate	436	39.1
bad	154	13.8
Ever visit psychiatrists due to own mental health issues		
yes	259	23.2
no	855	76.8
Ever visit psychotherapists due to own mental health issues		
yes	177	15.9
no	937	84.1
History of mental disorders in close family		
yes	235	21.1
no	879	78.9
History of mental disorders in close friends		
yes	116	10.4
no	998	89.6

**Table 2:** Public beliefs and perception of mental disorders among adults in Poland, 2025 (*n* = 1114).

Variable	*n*	%
Do You Agree that People with Mental Disorders (e.g., Depression) in Poland Receive the Same Level of Care as People with Physical Problems (e.g., Diabetes or Hypertension)?		
definitely yes	47	4.2
rather yes	169	15.2
rather no	342	30.7
definitely no	279	25.0
I do not know/difficult to tell	277	24.9
Do you think mental disorders can be effectively treated?		
definitely yes	192	17.2
rather yes	621	55.7
rather no	98	8.8
definitely no	26	2.3
I do not know/difficult to tell	177	15.9
To what extent do you agree with the statement that mental disorders are a sign of weakness?		
definitely yes	37	3.3
rather yes	144	12.9
rather no	307	27.6
definitely no	467	41.9
I do not know/difficult to tell	159	14.3
Do you think that people diagnosed with mental disorders are discriminated against in Poland?		
definitely yes	130	11.7
rather yes	466	41.8
rather no	207	18.6
definitely no	42	3.8
I do not know/difficult to tell	269	24.1
Do you think employers are afraid to hire people diagnosed with mental disorders?		
definitely yes	290	26.0
rather yes	532	47.8
rather no	72	6.5
definitely no	8	0.7
I do not know/difficult to tell	212	19.0

**Table 3:** Sociodemographic differences in public perception of mental disorder care in Poland.

Do You Agree that People with Mental Disorders (e.g., Depression) in Poland Receive the Same Level of Care as People with Physical Problems (e.g., Diabetes or Hypertension)?—“Rather No” or “Definitely No”						
			Bivariable Logistic Regression		Multivariable Logistic Regression	
Variable	%	*p*	OR (95%CI)	*p*	aOR (95%CI)	*p*
Gender						
female (n = 609)	58.6	**0.03**	1.29 (1.02–1.64)	0.03	1.22 (0.96–1.56)	0.1
male (n = 505)	52.3		Reference		Reference	
Age [years]						
18–34 (n = 260)	62.3	**0.02**	1.30 (0.89–1.90)	0.2		
35–49 (n = 321)	57.0		1.04 (0.73–1.50)	0.8		
50–64 (n = 342)	49.4		0.77 (0.54–1.10)	0.1		
65+ (n = 191)	56.0		Reference			
Place of residence						
rural area (n = 423)	50.8	**0.01**	Reference		Reference	
city below 20,000 residents (n = 143)	58.7		1.38 (0.94–2.02)	0.1	1.31 (0.88–1.94)	0.2
city from 20,000 to 99,999 residents (n = 218)	53.2		1.10 (0.79–1.53)	0.6	1.04 (0.74–1.45)	0.8
city from 100,000 to 499,999 residents (n = 187)	58.8		1.38 (0.98–1.96)	0.07	1.28 (0.90–1.83)	0.2
city ≥ 500,000 residents (n = 143)	67.1		1.98 (1.32–2.94)	<0.001	1.67 (1.11–2.51)	**0.01**
Having higher education						
yes (n = 508)	63.0	**<0.001**	1.73 (1.36–2.19)	<0.001	1.62 (1.26–2.07)	**<0.001**
no (n = 606)	49.7		Reference		Reference	
Occupational status						
active (n = 685)	56.6	0.4	1.10 (0.86–1.40)	0.4		
passive (n = 429)	54.3		Reference			
Married						
yes (n = 612)	54.4	0.3	0.89 (0.70–1.13)	0.3		
no (n = 502)	57.4		Reference			
Having children						
yes (n = 727)	53.5	**0.04**	Reference		Reference	
no (n = 387)	59.9		1.30 (1.01–1.67)	0.04	1.23 (0.95–1.59)	0.1
Self-reported economic status						
good (n = 524)	59.4	0.07	1.28 (0.89–1.84)	0.2		
moderate (n = 436)	52.3		0.96 (0.67–1.39)	0.8		
bad (n = 154)	53.2		Reference			
Ever visited a psychiatrist, due to own mental health issues						
yes (n = 259)	63.7	**0.003**	1.54 (1.15–2.05)	0.003	1.24 (0.91–1.69)	0.2
no (n = 855)	53.3		Reference		Reference	
Ever visited a psychotherapist, due to own mental health issues						
yes (n = 177)	59.3	0.3	1.19 (0.86–1.65)	0.3		
no (n = 937)	55.1		Reference			
History of mental disorders in close family						
yes (n = 235)	66.0	**<0.001**	1.72 (1.27–2.32)	<0.001	1.56 (1.13–2.14)	**0.01**
no (n = 879)	53.0		Reference		Reference	
History of mental disorders in close friends						
yes (n = 116)	69.8	**0.001**	1.96 (1.30–2.97)	0.001	1.63 (1.05–2.53)	**0.03**
no (n = 998)	54.1		Reference		Reference	

**Table 4:** Sociodemographic differences in public perception of mental disorder treatment.

Do You Think Mental Disorders Can Be Effectively Treated?—“Rather Yes” or “Definitely Yes”						
			Bivariable Logistic Regression		Multivariable Logistic Regression	
Variable	%	*p*	OR (95%CI)	*p*	aOR (95%CI)	*p*
Gender						
female (n = 609)	75.0	0.1	1.26 (0.97–1.64)	0.1		
male (n = 505)	70.5		Reference			
Age [years]						
18–34 (n = 260)	75.8	**0.01**	1.08 (0.70–1.66)	0.7		
35–49 (n = 321)	66.0		0.67 (0.45–1.00)	0.05		
50–64 (n = 342)	76.6		1.13 (0.75–1.70)	0.6		
65+ (n = 191)	74.3		Reference			
Place of residence						
rural area (n = 423)	71.2	0.6	Reference			
city below 20,000 residents (n = 143)	70.6		0.98 (0.64–1.48)	0.9		
city from 20,000 to 99,999 residents (n = 218)	73.9		1.15 (0.79–1.65)	0.5		
city from 100,000 to 499,999 residents (n = 187)	74.9		1.21 (0.82–1.79)	0.3		
city ≥ 500,000 residents (n = 143)	76.9		1.35 (0.87–2.10)	0.2		
Having higher education						
yes (n = 508)	77.6	**0.002**	1.54 (1.18–2.02)	0.002	1.47 (1.11–1.94)	**0.01**
no (n = 606)	69.1		Reference		Reference	
Occupational status						
active (n = 685)	73.9	0.4	1.12 (0.86–1.47)	0.4		
passive (n = 429)	71.6		Reference			
Married						
yes (n = 612)	73.9	0.5	1.10 (0.85–1.44)	0.5		
no (n = 502)	71.9		Reference			
Having children						
yes (n = 727)	74.0	0.3	1.16 (0.88–1.53)	0.3		
no (n = 387)	71.1		Reference			
Self-reported economic status						
good (n = 524)	77.7	**0.004**	1.62 (1.09–2.41)	0.02	1.60 (1.06–2.40)	**0.02**
moderate (n = 436)	69.0		1.04 (0.70–1.55)	0.8	1.04 (0.69–1.55)	0.9
bad (n = 154)	68.2		Reference		Reference	
Ever visited a psychiatrist, due to own mental health issues						
yes (n = 259)	83.4	**<0.001**	2.17 (1.52–3.11)	<0.001	1.89 (1.24–2.87)	**0.003**
no (n = 855)	69.8		Reference		Reference	
Ever visited a psychotherapist, due to own mental health issues						
yes (n = 177)	80.8	**0.01**	1.68 (1.12–2.50)	0.01	0.96 (0.60–1.54)	0.9
no (n = 937)	71.5		Reference		Reference	
History of mental disorders in close family						
yes (n = 235)	82.1	**<0.001**	1.92 (1.33–2.76)	<0.001	1.63 (1.11–2.38)	**0.01**
no (n = 879)	70.5		Reference		Reference	
History of mental disorders in close friends						
yes (n = 116)	85.3	**0.002**	2.32 (1.36–3.95)	0.002	1.77 (1.01–3.08)	**0.04**
no (n = 998)	71.5		Reference		Reference	

**Table 5:** Sociodemographic differences in public perception of mental disorders as a sign of weakness.

Do You Agree with the Statement that Mental Disorders Are a Sign of Weakness?—“Rather Yes” or “Definitely Yes”						
			Bivariable Logistic Regression		Multivariable Logistic Regression	
Variable	%	*p*	OR (95%CI)	*p*	aOR (95%CI)	*p*
Gender						
female (n = 609)	12.3	**<0.001**	Reference		Reference	
male (n = 505)	21.0		1.89 (1.37–2.61)	<0.001	1.88 (1.36–2.61)	**<0.001**
Age [years]						
18–34 (n = 260)	17.3	0.8	0.97 (0.59–1.58)	0.9		
35–49 (n = 321)	15.9		0.87 (0.54–1.41)	0.6		
50–64 (n = 342)	14.9		0.81 (0.50–1.30)	0.4		
65+ (n = 191)	17.8		Reference			
Place of residence						
rural area (n = 423)	16.3	0.09	1.27 (0.74–2.20)	0.4	1.26 (0.72–2.20)	0.4
city below 20,000 residents (n = 143)	13.3		1.00 (0.51–1.98)	0.9	1.02 (0.51–2.02)	0.9
city from 20,000 to 99,999 residents (n = 218)	22.0		1.84 (1.03–3.29)	0.04	1.82 (1.01–3.27)	**0.04**
city from 100,000 to 499,999 residents (n = 187)	13.9		1.05 (0.56–1.99)	0.9	1.06 (0.56–2.02)	0.9
city ≥ 500,000 residents (n = 143)	13.3		Reference		Reference	
Having higher education						
yes (n = 508)	14.2	0.09	0.75 (0.54–1.04)	0.09		
no (n = 606)	18.0		Reference			
Occupational status						
active (n = 685)	16.1	0.8	0.97 (0.70–1.34)	0.8		
passive (n = 429)	16.6		Reference			
Married						
yes (n = 612)	16.2	0.9	0.99 (0.72–1.36)	0.9		
no (n = 502)	16.3		Reference			
Having children						
yes (n = 727)	16.9	0.4	1.16 (0.82–1.62)	0.4		
no (n = 387)	15.0		Reference			
Self-reported economic status						
good (n = 524)	18.1	**0.03**	0.88 (0.56–1.38)	0.6	0.92 (0.58–1.45)	0.7
moderate (n = 436)	12.6		0.57 (0.35–0.93	0.02	0.60 (0.36–0.97)	**0.04**
bad (n = 154)	20.1		Reference		Reference	
Ever visited a psychiatrist, due to own mental health issues						
yes (n = 259)	17.4	0.6	1.11 (0.77–1.61)	0.6		
no (n = 855)	15.9		Reference			
Ever visited a psychotherapist, due to own mental health issues						
yes (n = 177)	20.9	0.07	1.46 (0.97–2.18)	0.07		
no (n = 937)	15.4		Reference			
History of mental disorders in close family						
yes (n = 235)	17.0	0.7	1.07 (0.73–1.58)	0.7		
no (n = 879)	16.0		Reference			
History of mental disorders in close friends						
yes (n = 116)	19.0	0.4	1.24 (0.75–2.02)	0.4		
no (n = 998)	15.9		Reference			

**Table 6:** Sociodemographic differences in public perception of people with mental disorders as a discriminated group in Poland.

Do You Think that People Diagnosed with Mental Disorders Are Discriminated Against in Poland? —“Rather Yes” or “Definitely Yes”						
			Bivariable Logistic Regression		Multivariable Logistic Regression	
Variable	%	*p*	OR (95%CI)	*p*	aOR (95%CI)	*p*
Gender						
female (n = 609)	60.3	**<0.001**	1.83 (1.44–2.32)	<0.001	1.74 (1.36–2.22)	**<0.001**
male (n = 505)	45.3		Reference		Reference	
Age [years]						
18–34 (n = 260)	55.8	0.2	1.20 (0.82–1.74)	0.4		
35–49 (n = 321)	57.0		1.26 (0.88–1.80)	0.2		
50–64 (n = 342)	49.7		0.94 (0.66–1.34)	0.7		
65+ (n = 191)	51.3		Reference			
Place of residence						
rural area (n = 423)	51.1	0.7	0.80 (0.55–1.17)	0.2		
city below 20,000 residents (n = 143)	56.6		1.00 (0.63–1.60)	0.9		
city from 20,000 to 99,999 residents (n = 218)	52.8		0.86 (0.56–1.31)	0.5		
city from 100,000 to 499,999 residents (n = 187)	55.1		0.94 (0.61–1.46)	0.8		
city ≥ 500,000 residents (n = 143)	56.6		Reference			
Having higher education						
yes (n = 508)	57.3	**0.02**	1.32 (1.04–1.68)	0.02	1.28 (1.00–1.64)	0.05
no (n = 606)	50.3		Reference		Reference	
Occupational status						
active (n = 685)	53.9	0.8	1.04 (0.82–1.32)	0.8		
passive (n = 429)	52.9		Reference			
Married						
yes (n = 612)	50.8	**0.04**	0.79 (0.62–0.99)	0.04	0.86 (0.67–1.10)	0.2
no (n = 502)	56.8		Reference		Reference	
Having children						
yes (n = 727)	52.4	0.3	0.88 (0.69–1.13)	0.3		
no (n = 387)	55.6		Reference			
Self-reported economic status						
good (n = 524)	55.9	**0.01**	Reference		Reference	
moderate (n = 436)	47.9		0.73 (0.56–0.94)	0.01	0.74 (0.57–0.97)	**0.03**
bad (n = 154)	61.0		1.24 (0.86–1.78)	0.3	1.26 (0.86–1.85)	0.2
Ever visited a psychiatrist, due to own mental health issues						
yes (n = 259)	62.5	**<0.001**	1.62 (1.22–2.16)	<0.001	1.09 (0.77–1.54)	0.6
no (n = 855)	50.8		Reference		Reference	
Ever visited a psychotherapist, due to own mental health issues						
yes (n = 177)	66.7	**<0.001**	1.92 (1.37–2.69)	<0.001	1.29 (0.86–1.93)	0.2
no (n = 937)	51.0		Reference		Reference	
History of mental disorders in close family						
yes (n = 235)	70.2	**<0.001**	2.45 (1.80–3.34)	<0.001	2.13 (1.53–2.96)	**<0.001**
no (n = 879)	49.0		Reference		Reference	
History of mental disorders in close friends						
yes (n = 116)	72.4	**<0.001**	2.49 (1.63–3.81)	<0.001	2.00 (1.27–3.14)	**0.003**
no (n = 998)	51.3		Reference		Reference	

**Table 7:** Sociodemographic differences in public perception of the position of people with mental disorders in the labor market.

Do You Think Employers Are Afraid to Hire People Diagnosed with Mental Disorders?—“Rather Yes” or “Definitely Yes”						
			Bivariable Logistic Regression		Multivariable Logistic Regression	
Variable	%	*p*	OR (95%CI)	*p*	aOR (95%CI)	*p*
Gender						
female (n = 609)	74.7	0.4	1.11 (0.85–1.45)	0.4		
male (n = 505)	72.7		Reference			
Age [years]						
18–34 (n = 260)	68.8	**0.02**	Reference		Reference	
35–49 (n = 321)	71.3		1.13 (0.79–1.61)	0.5	1.12 (0.76–1.64)	0.6
50–64 (n = 342)	75.7		1.41 (0.99–2.03)	0.06	1.55 (1.01–2.36)	**0.04**
65+ (n = 191)	81.2		1.95 (1.25–3.05)	0.003	2.18 (1.30–3.64)	**0.003**
Place of residence						
rural area (n = 423)	71.6	0.5	1.02 (0.67–1.54)	0.9		
city below 20,000 residents (n = 143)	75.5		1.24 (0.73–2.10)	0.4		
city from 20,000 to 99,999 residents (n = 218)	77.1		1.35 (0.84–2.18)	0.2		
city from 100,000 to 499,999 residents (n = 187)	75.4		1.23 (0.75–2.02)	0.4		
city ≥ 500,000 residents (n = 143)	71.3		Reference			
Having higher education						
yes (n = 508)	78.5	**<0.001**	1.58 (1.20–2.08)	<0.001	1.65 (1.25–2.19)	**<0.001**
no (n = 606)	69.8		Reference		Reference	
Occupational status						
active (n = 685)	72.3	0.1	0.81 (0.62–1.07)	0.1		
passive (n = 429)	76.2		Reference			
Married						
yes (n = 612)	75.2	0.2	1.17 (0.90–1.53)	0.3		
no (n = 502)	72.1		Reference			
Having children						
yes (n = 727)	75.8	**0.04**	1.34 (1.02–1.77)	0.04	1.13 (0.81–1.58)	0.5
no (n = 387)	70.0		Reference		Reference	
Self-reported economic status						
good (n = 524)	76.3	**0.01**	0.85 (0.55–1.31)	0.5	0.80 (0.51–1.25)	0.3
moderate (n = 436)	68.8		0.58 (0.37–0.90)	0.02	0.54 (0.35–0.85)	**0.01**
bad (n = 154)	79.2		Reference		Reference	
Ever visited a psychiatrist, due to own mental health issues						
yes (n = 259)	75.7	0.4	1.14 (0.83–1.57)	0.4		
no (n = 855)	73.2		Reference			
Ever visited a psychotherapist, due to own mental health issues						
yes (n = 177)	76.8	0.3	1.21 (0.83–1.77)	0.3		
no (n = 937)	73.2		Reference			
History of mental disorders in close family						
yes (n = 235)	80.9	**0.006**	1.65 (1.16–2.36)	0.006	1.60 (1.11–2.30)	**0.01**
no (n = 879)	71.9		Reference		Reference	
History of mental disorders in close friends						
yes (n = 116)	84.6	**0.01**	1.92 (1.15–3.21)	0.01	2.00 (1.18–3.38)	**0.01**
no (n = 998)	72.6		Reference		Reference	

## Data Availability

The raw data supporting the conclusions of this article will be made available by the authors on request.

## Supplementary Materials

The following supporting information can be downloaded at: https://www.mdpi.com/article/10.3390/jcm14134586/s1, Supplementary Materia S1. Study questionnaire.
