# Supplementary material for: Public Beliefs and Perception of Mental Disorders in Poland—A 2025 Nationwide Cross-Sectional Survey
Source: J Clin Med. 2025 Jun 28;14(13):4586. doi: 10.3390/jcm14134586 (PMC12250092; doi:10.3390/jcm14134586)
Supplement: Supplementary file 1 [file jcm-14-04586-s001.zip › jcm-3717196-supplementary.pdf]

Supplementary material S1. Study Questionnaire

1/ Do you agree that people with mental disorders (e.g., depression) in Poland receive the same level of care as people with physical problems (e.g., diabetes or hypertension)?

- definitely yes
- rather yes
- rather no
- definitely no
- I do not know / difficult to tell

2/ Do you think mental disorders can be effectively treated?

- definitely yes
- rather yes
- rather no
- definitely no
- I do not know / difficult to tell

3/ To what extent do you agree with the statement that mental disorders are a sign of weakness?

- definitely yes
- rather yes
- rather no
- definitely no
- I do not know / difficult to tell

4/ Do you think that people diagnosed with mental disorders are discriminated in Poland?

- definitely yes
- rather yes
- rather no
- definitely no
- I do not know / difficult to tell

5/ Do you think employers are afraid to hire people diagnosed with mental disorders?

- definitely yes
- rather yes
- rather no
- definitely no
- I do not know / difficult to tell

Sociometric questions

1. What is your gender?

- woman
- man

2. How old are you?

________________________[age in years]

3. What is the size of the town you live in?

- village
- small town (up to 20,000 inhabitants)
- medium-sized city (20,000 to 99,000 inhabitants)
- large city (between 100,000 and 500,000 inhabitants)
- large city (more than 500,000 inhabitants)

__________please insert your postal code

4. What is your current education (most recently completed school)?

- primary or lower secondary school
- vocational
- secondary
- post-secondary (po. szkoła policealna)
- bachelor degree
- completed university degree

5. How would you rate your household financial situation?

- good
- moderate
- bad

7. What is your current professional status?

- I work under an employment contract
- I work on a civil contract basis
- I am self-employed
- unemployed
- pensioner
- pupil or student
- I am in charge of housekeeping
- other (provide details)

8. What is your marital status?

- single
- married
- in an informal relationship
- other (provide details)

9. Do you have children under 18 living with you?

- yes
- no

10. In which province do you live?

- Dolnośląskie
- Kujawsko-Pomorskie
- Lublin
- Lubuskie
- Lodz
- Małopolskie
- Mazowsze
- Opolskie
- Podkarpackie
- Podlaskie
- Pomeranian
- Śląskie
- Świętokrzyskie
- Warminsko-Mazurskie
- Wielkopolskie
- Zachodniopomorskie
